# Is the practice of colorectal cancer screening questionable after the NordICC trial was published?

**DOI:** 10.1002/ctm2.1365

**Published:** 2023-10-04

**Authors:** Martin C. S. Wong, Junjie Huang, Peter S. Liang

**Affiliations:** ^1^ Centre for Health Education and Health Promotion JC School of Public Health and Primary Care Faculty of Medicine The Chinese University of Hong Kong Hong Kong China; ^2^ NYU Grossman School of Medicine New York New York USA

**Keywords:** cancer, colorectal, screening

1

Worldwide, the incidence of colorectal cancer (CRC) increased substantially in various countries, which could be attributed to the rising rate of lifestyle, metabolic, and environmental risk factors due to globalization, urbanization and westernization.^1^, ^2^ Nevertheless, the value of population‐based, CRC screening has been challenged by recent evidence.^3^ It was opined that CRC screening may offer limited value at the population level, mainly due to its modest impact on decreased CRC mortality based on findings from the NordICC trial, the first pragmatic, randomised controlled trial of colonoscopy screening.^4^ According to the landmark study, which included data from 84,585 healthy subjects aged 55–64 years recruited from three European countries between 2009 and 2014, the risk ratio (0.90, 95% confidence interval [CI] 0.64–1.16) and absolute risk reduction (−0.03, 95% CI −0.11–0.05) of mortality from CRC were not statistically significant. This finding has initiated an update in a clinical practice guideline—which now recommends CRC screening among people aged 50−79 years with a high 15‐year risk of developing CRC (e.g. those with risk > 3%) only—but not the general population at lower risk.^5^ Whilst we agree that the death rate is one of the principal considerations, we believe this topic should be revisited from another perspective.

First, CRC screening meets the vast majority, if not all, criteria of screening proposed by Wilson and Jungner (WHO), which remains a widely recognized standard. Screening may lead to the reduction of CRC incidence by around 18%.^4^ The use of faecal immunochemical tests (FITs) has been proven successful to reduce the incidence of metastatic CRC by approximately 70% after the first round.^6^ The IMPATTO study from Italy has shown that individuals who attended FIT screening had a significantly higher risk of stage I CRC (relative risk ratio [RRR]: 2.04, 95% CI 1.84–2.46) but much lower risk and incidence rate ratio (IRR) of stage IV CRC (RRR: 0.77, 95% CI 0.69–0.87; IRR: 0.7, 95% CI 0.6–0.9). This has been attributed to the fact that repeated FIT screening at regular intervals enables the detection and removal of adenomatous polyps, which could develop into CRC.^7^ These findings were also replicated in large‐scale observational studies, demonstrating a net reduction of incidence after 7−8 years of FIT screening.

Secondly, one caution in the interpretation of the NordICC trial includes its subject recruitment being conducted in European countries only. CRC mortality is highly influenced by the accessibility of healthcare, technological advances of therapies available, the quality of care provided by medical professionals, and treatment‐related policies that are country‐specific. One might argue that other regions with poorer treatment resources could have a bigger difference in CRC mortality rate between the colonoscopy screening group and the control group.

What is the societal cost of missing early‐stage CRC and thus early treatment? The cost of managing metastatic CRC imposes a substantial economic burden on patients, caregivers, populations, and the healthcare system.^8^ From a systematic review of studies that included the medical cost of systemic therapy, the cost of treating one metastatic CRC was estimated at US$300 000, a prohibitively high health care expenditure in most countries.^8^ This does not even include the costs associated with loss of productivity, job absenteeism, early retirement due to the disease, and various damages of the disease stigma and therapies on one's psychological and social well‐being. Pre‐Medicare CRC screening with a 70% coverage rate has been estimated to reduce Medicare spending by US$14 billion in 2050.

Whilst colonoscopy screening is associated with potential harms due to its invasive nature, the pooled overall serious adverse event rate was only 2.8 per 1000 procedures^9^ and this was even lower if FIT is used for screening.

Will shared decision‐making between general practitioners and prospective screening participants cause a prolonged consultation with little value for money? It depends on whether the physicians have chosen the right patient as part of anticipatory care. There is no gold standard to predict which preventive care advice will offer the most optimal patient benefits, as one may never know the receptiveness of patients to a wide array of different options. Besides, could physicians easily judge whether one successful smoking cessation case is superior to an additional influenza vaccine received due to opportunistic care? By all means, it is inappropriate to recommend CRC screening for all clinical consultations, but a large proportion of the population would benefit from screening, especially those with risk factors such as smoking and a family history of CRC.

Hence, CRC screening remains an excellent opportunity for preventive care if patient outcomes that matter, other than mortality, are considered. A previous population‐based survey based on the Health Belief Model in more than 1000 community‐dwelling subjects showed that individuals who received physician recommendations were 23 times more likely to have attended a CRC screening test.^10^ Understandably, the chance of success is higher for CRC screening than for other opportunistic care that aims to modify long‐standing behaviour like sedentary lifestyle habits and alcohol addiction, which are notoriously difficult to change. Nevertheless, we agree that using a risk‐stratified strategy will be a sensible approach because we have to utilize resources in a cost‐effective manner.

It is time for clinicians to consider what preventive care should be offered in their clinical practice—at the right time, with the right patient, and in the context of various preventive care options. Currently, there has been little research that compared the cost‐effectiveness and efficiency of various preventive advice in clinical practice—and importantly how physicians should select them. It is high time to devise and formulate guidelines and consensus recommendations for reference by our medical professionals.

## CONFLICT OF INTEREST STATEMENT

Martin C. S. Wong is the honorary medical advisor of GenieBiome Ltd. and an honorary consultant of SunRise Ltd. He is an advisory committee member of Pfizer; an external expert of GlaxoSmithKline Limited; a member of the advisory board of Astra‐Zeneca and has been paid consultancy fees for providing advice on research. Junjie Huang did not declare any conflict of interest. Peter S. Liang has received research support from Epigenomics and Freenome and serves on the advisory board for Guardant Health.
